# Supplementary material for: HIVprotI: an integrated web based platform for prediction and design of HIV proteins inhibitors
Source: J Cheminform. 2018 Mar 9;10:12. doi: 10.1186/s13321-018-0266-y (PMC5845081; doi:10.1186/s13321-018-0266-y)
Supplement: Supplementary file 2 — Additional file 2. Source code of HIVProtI web server. [file 13321_2018_266_MOESM2_ESM.zip › HIVprotI_Source-code/hivprotI/weka-3-6-6/doc/weka/attributeSelection/package-frame.html]

weka.attributeSelection


weka.attributeSelection

Interfaces 
  
*AttributeEvaluator*
  
*AttributeTransformer*
  
*ErrorBasedMeritEvaluator*
  
*RankedOutputSearch*
  
*StartSetHandler*
  
*SubsetEvaluator*

Classes 
  
ASEvaluation
  
ASSearch
  
AttributeSelection
  
AttributeSetEvaluator
  
BestFirst
  
CfsSubsetEval
  
CheckAttributeSelection
  
ChiSquaredAttributeEval
  
ClassifierSubsetEval
  
ConsistencySubsetEval
  
CostSensitiveASEvaluation
  
CostSensitiveAttributeEval
  
CostSensitiveSubsetEval
  
ExhaustiveSearch
  
FilteredAttributeEval
  
FilteredSubsetEval
  
GainRatioAttributeEval
  
GeneticSearch
  
GreedyStepwise
  
HoldOutSubsetEvaluator
  
InfoGainAttributeEval
  
LatentSemanticAnalysis
  
LFSMethods
  
LinearForwardSelection
  
OneRAttributeEval
  
PrincipalComponents
  
RaceSearch
  
RandomSearch
  
Ranker
  
RankSearch
  
ReliefFAttributeEval
  
ScatterSearchV1
  
SubsetSizeForwardSelection
  
SVMAttributeEval
  
SymmetricalUncertAttributeEval
  
UnsupervisedAttributeEvaluator
  
UnsupervisedSubsetEvaluator
  
WrapperSubsetEval
